# Eps15R is required for bone morphogenetic protein signalling and differentially compartmentalizes with Smad proteins

**DOI:** 10.1098/rsob.120060

**Published:** 2012-04

**Authors:** Elizabeth M. Callery, Chong Yon Park, Xin Xu, Haitao Zhu, James C. Smith, Gerald H. Thomsen

**Affiliations:** 1 Department of Medicine, University of Cambridge, PO Box 157, Addenbrooke's Hospital, Cambridge CB2 0QQ, UK; 2 MRC National Institute for Medical Research, The Ridgeway, London NW7 1AA, UK; 3 Department of Biochemistry and Cell Biology and Center for Developmental Genetics, Stony Brook University, Stony Brook, NY 11794-5215, USA

**Keywords:** Eps15R, Smad, bone morphogenetic protein, endocytosis, bimolecular fluorescence complementation

## Abstract

Transforming growth factor β superfamily members signal through Smad transcription factors. Bone morphogenetic proteins (BMPs) act via Smads 1, 5 and 8 and TGF-βs signal through Smads 2 and 3. The endocytic adaptor protein Eps15R, or ‘epidermal growth factor (EGF) receptor pathway substrate 15-related protein’ is a component of EGF signal transduction, mediating internalization of the EGF receptor. We show that it interacts with Smad proteins, is required for BMP signalling in animal caps and stimulates Smad1 transcriptional activity. This function resides in the Asp-Pro-Phe motif-enriched ‘DPF domain’ of Eps15R, which activates transcription and antagonizes Smad2 signalling. In living cells, Eps15R segregates into spatially distinct regions with different Smads, indicating an unrecognized level of Smad compartmentalization.

## 2. Introduction

Members of the transforming growth factor β (TGF-β) superfamily signal through transmembrane receptors comprising type I and type II serine/threonine kinases, and the two main TGF-β subfamilies, the bone morphogenetic proteins (BMPs) and the TGF-βs, activins and nodals, activate distinct combinations of type I and type II receptors [1]. These activate different Smad transcription factors: BMPs activate Smads 1, 5 and 8, and TGF-βs activate Smads 2 and 3. These receptor-regulated Smads, together with their common partner Smad4, orchestrate ligand-specific transcriptional responses [2].

The BMPs function in embryonic axis formation, neural development and adult tissue homeostasis. Pathological consequences of BMP dysregulation include birth defects and cancer: an understanding of BMP signalling is thus of clinical as well as biological significance [3,4].

Endocytic components help control the response to paracrine signalling, in part by regulating cell-surface receptor levels [5–9]. Eps15R is a member of the Eps15 homology domain (EH domain) family [10], whose members facilitate clathrin-mediated endocytosis via interactions with the clathrin adaptor protein AP2 [11]. Eps15R functions in epidermal growth factor (EGF) signalling, where it is required for receptor internalization [12,13]. Here, we show that Eps15R interacts with Smad1 and is required for BMP signalling but not for activin or fibroblast growth factor (FGF) responsiveness. By visualizing Eps15R–Smad complexes in living cells, we show, remarkably, that Eps15R segregates into distinct subcellular pools when paired with either Smad1 or Smad2.

## 3. Results and discussion

### 3.1. Eps15R interacts with Smad1

*Xenopus* embryos provide a powerful model system to analyse BMP signalling. To identify BMP pathway modulators, we performed a yeast two-hybrid screen using Smad1 as bait and a *Xenopus* cDNA library as prey. We obtained a fragment of *Xenopus laevis* Eps15R and then isolated a full-length version from a *Xenopus* cDNA library.

**Figure 1.**
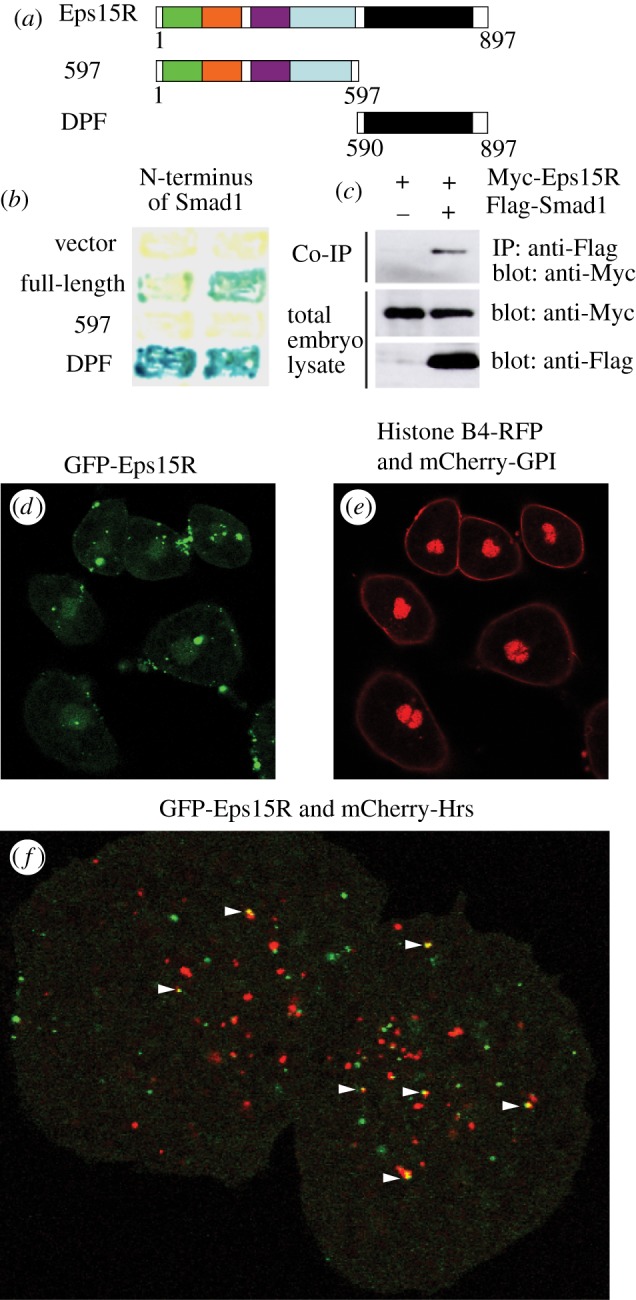
The endocytic adaptor protein Eps15R interacts with Smad1. (*a*) *Xenopus* Eps15R and deletion constructs. The N-terminal portion contains three Eps15 homology (EH) domains (green, orange and purple), followed by a coiled coil domain (blue) and carboxy-terminal DPF tripeptide repeat domain (black). (*b*) Eps15R binds to Smad1 in the yeast two-hybrid assay, as detected by β-galactosidase activity. Interaction requires the DPF domain of Eps15R. (*c*) Eps15R and Smad1 interact in *Xenopus* embryos. Embryos were injected with Myc-tagged full-length Eps15R mRNA alone or with Flag-tagged Smad1 mRNA. Lysates were immunoprecipitated (IP) with anti-FLAG antibodies and then western blotted with anti-Myc antibodies. Whole embryo lysates were immunoblotted with anti-Myc and anti-FLAG antibodies. (*d*) GFP-Eps15R is enriched in bright punctate foci located both juxta-membraneously and deeper within the cytoplasm of *Xenopus* animal cap cells. Lower fluorescence levels are found in the nucleus. (*e*) Histone B4-RFP and mCherry-GPI expression in cells shown in (*d*). (*f*) Limited co-localization of GFP-Eps15R and mCherry-Hrs 1 h after stimulation with 100 ng ml^–1^ BMP4/7.

*Xenopus Eps15R* encodes a protein of 897 amino acids that shares 73 per cent amino acid identity with human and mouse Eps15R. Like its mammalian orthologues, *Xenopus* Eps15R contains three copies of an EH domain and a coiled-coil domain that mediates protein dimerization [14]. At their C-termini, *Xenopus* and mammalian Eps15Rs contain a series of aspartate-proline-phenylalanine (DPF) tripeptide repeats, known as the DPF domain [10] (figure 1*a*). We found that Eps15R interacts with the MH1 domain of Smad1 through the DPF domain both in yeast by two-hybrid assay (figure 1*b*) and in *Xenopus* embryos by co-immunoprecipitation (figure 1*c*).

*Eps15R* is widely expressed during *Xenopus* development, and is enriched in neural tissue and mesodermal derivatives such as somites (see electronic supplementary material,  figure S1). We examined its protein localization in *Xenopus* ectodermal cells using a GFP-tagged version of Eps15R after confirming the functionality of this protein (see electronic supplementary material, figure S2). There was weak nuclear fluorescence and strong fluorescence in punctate foci at the cell surface, consistent with the reported distribution of mammalian Eps15R (figure 1*d,e*). Some foci were intracellular, suggesting that Eps15R is present in a subset of endocytic vesicles. Eps15 interacts with the endosomal marker Hrs [15,16]; so we asked whether Eps15R co-localizes with this protein. A few vesicles were positive for both proteins, in unstimulated cells (data not shown) and in cells treated with BMP4/7 (figure 1*f*). Such overlap was rare, suggesting that Eps15R resides predominantly in a compartment distinct from Hrs.

### 3.2. Eps15R is a specific modulator of bone morphogenetic protein signalling

**Figure 2.**
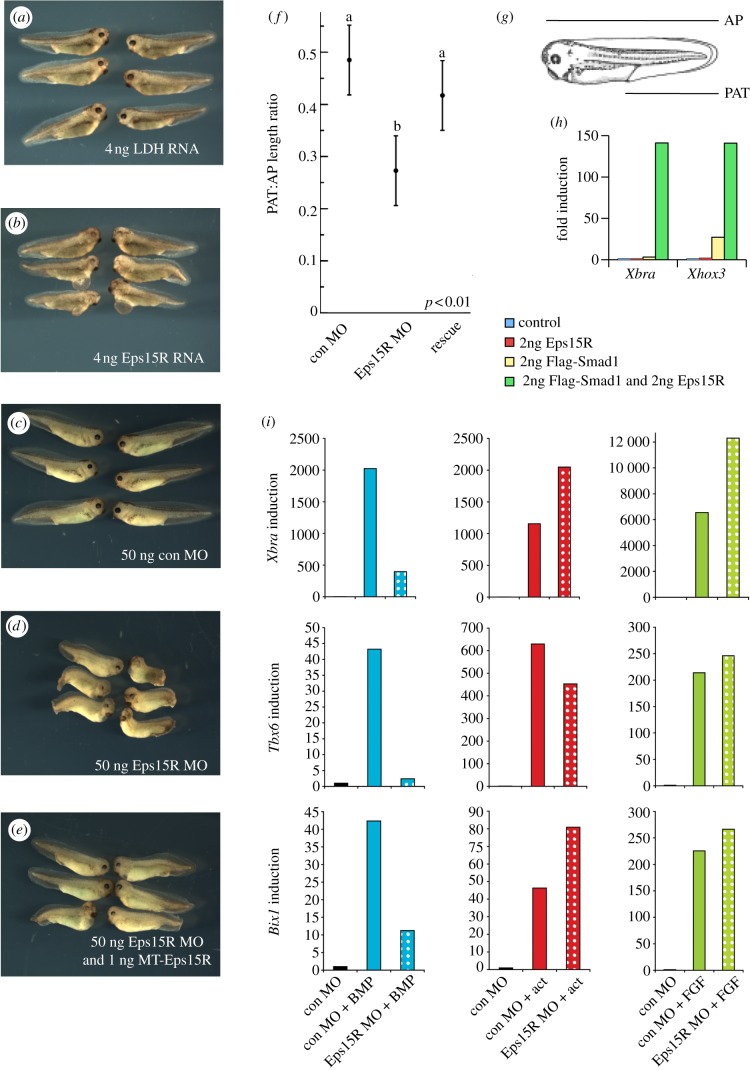
Eps15R enhances Smad1 signalling and is required for transcription in response to BMP signalling. (*a,b*) Over-expression of a control RNA encoding lactate dehydrogenase (LDH) does not impair development (*a*), whereas over-expression of Eps15R RNA causes defects in head and anterior mesoderm (*b*). (*c–e*) Phenotypes of embryos injected with an antisense MO oligonucleotide targeting Eps15R. (*c*) Controls. (*d*) Embryos injected with Eps15R MO exhibit shortened axes and ventrolateral defects. (*e*) The phenotype in (*d*) is significantly rescued by co-injection of MT-Eps15R RNA. (*f*) Morphometric analysis of PAT : AP length ratio in these embryos. Measurements were subjected to ANOVA and Tukey's test for least significant difference. (*g*) Diagrammatic depiction of the PAT and AP measurements. (*h*) Expression of BMP targets *Xhox3* and *Xbra* is elevated when Eps15R is co-expressed with Flag-Smad1 in the animal cap assay; caps were harvested at NF11.5. (*i*) Eps15R MO inhibits transcription of BMP-responsive genes in animal caps treated with 100 ng ml^–1^ BMP4/7 heterodimers and harvested at NF11.5, yet there is no decrease in activation of these genes in response to 100 ng ml^–1^ FGF4 (FGF) or 10 ng ml^–1^ activin (act). Fold induction was calculated relative to control cap levels, and samples were normalized to the expression of *ornithine decarboxylase*.

Over-expression of Eps15R RNA caused defects of the eye and anterior mesendoderm (figure 2*a*,*b*). Loss of function was achieved using a translation-blocking antisense morpholino (MO). Morphant embryos had short axes, eye defects and swelling around the heart cavity (figure 2*c*,*d*). Co-injection of Myc-tagged (MT)-Eps15R RNA reduced the severity of defects (figure 2*e*). Whereas 81 per cent (*n* = 26) of Eps15R morphants had curved axes, such defects were only observed in 17 per cent (*n* = 24) of rescued embryos and 8 per cent (*n* = 24) of embryos injected with control MO. We quantitated the degree of axial rescue using the morphometric ratio of post-anal tail length : anteroposterior length (PAT/AP length ratio; figure 2*f*,*g*). Whereas this measure was decreased in Eps15R morphants (*p* < 0.01), there was no significant difference between the control and rescue groups, indicating a successful rescue of this phenotype (figure 2*f*).

The interaction between Eps15R and Smad1 suggests that Eps15R is involved in BMP signalling. Over-expression of Eps15R in animal caps did not activate BMP-responsive genes (figure 2*h*). However, co-expression of Eps15R with Smad1 enhances the ability of this BMP signal transducer to activate targets such as *Xbra* [17–21] and *Xhox3* [22] (figure 2*h*). The GFP-Eps15R construct also synergizes with Smad1 to upregulate these genes, confirming that the fusion protein we used in our localization assays (figure 1) retains functional activity (see electronic supplementary material, figure S2). The results of these synergy experiments suggest that Eps15 family proteins function in BMP signalling in addition to their role in EGF signalling. Further support for this comes from our discovery that the Eps15R MO significantly reduced BMP4/7-induced expression of the target genes *Xbra*, *Tbx6*, *Bix1* and *Vent1* in animal caps [23] (figure 2*i* and data not shown). The inhibitory effect of Eps15R depletion on BMP signalling is specific; neither activin nor FGF signalling was significantly reduced by loss of Eps15R (figure 2*i*).

### 3.3. The DPF domain transactivates gene expression and mimics bone morphogenetic protein activation

**Figure 3.**
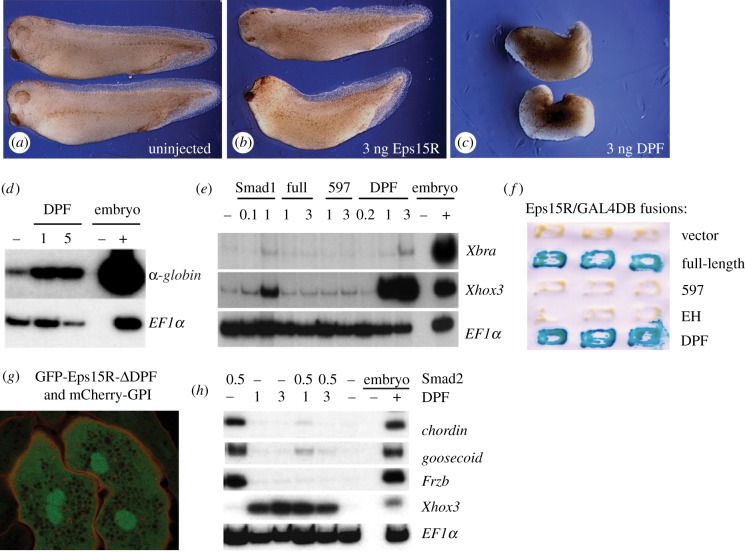
The DPF domain transactivates gene expression and differentially modulates Smad signalling. (*a*–*c*) Effects of over-expression of full-length Eps15R, or the DPF domain alone, on *Xenopus* development. (*a*) Uninjected embryos. (*b*) Embryos injected with 3 ng RNA encoding Eps15R. (*c*) Embryos injected with 3 ng RNA encoding the Eps15R DPF domain. (*d*) RT-PCR showing *globin* induction by the DPF domain in NF21 animal caps. (*e*) The DPF domain mimics the activity of Smad1 in the induction of *Xbra* and *Xhox3* in NF11 gastrula caps but the full-length Eps15R lacks this ability. (*f*) Both full length Eps15R and the DPF domain can transactivate transcription in the yeast assay. (*g*) The cytoplasmic localization of GFP-Eps15R-*Δ*DPF is disrupted, displaying a diffuse, reticulated cytoplasmic distribution, in contrast to the punctate cytoplasmic localization of the full-length version shown in figure 1*d*. Nuclear enrichment is retained in the absence of the DPF domain. (*h*) The DPF domain antagonizes the ability of Smad2 to induce expression of genes such as *chordin*, *goosecoid* and *Frzb* in NF11 gastrula animal caps, which instead activates the ventral marker *Xhox3*.

The DPF domain is required for the interaction of Eps15R with Smad1 (figure 1*b*) and it also binds AP2, which recruits clathrin to the cell surface [24]. We tested the activity of the DPF domain by injecting embryos with RNA encoding this protein region. Development appeared significantly perturbed by injection of 3 ng mRNA encoding the DPF domain, relative to an equivalent amount of full-length RNA (figure 3*a–c*), although a precise quantitative comparison of the effects of the RNAs would require equivalent molar amounts to be injected. The embryos appeared ventralized, consistent with the ability of the DPF domain to induce expression of the ventral marker α-*globin* in animal caps (figure 3*d*). The DPF domain of Esp15R mimics Smad1-mediated gene induction, upregulating the expression of *Xbra* and *Xhox3* in animal caps (figure 3*e*), whereas neither the full-length protein nor the N-terminal 597 domain possesses this ability. It is possible, in a manner analogous to Smad proteins [25], that intramolecular interactions between the N-terminal EH domains and the DPF repeats inhibit the function of the full-length protein, and that these do not occur when the DPF domain is expressed alone.

The ability of the DPF domain to mediate transcription was confirmed by a yeast activation assay (figure 3*f*), which showed that full-length *Xenopus* Eps15R has the ability to transactivate gene expression, and that this activity resides within the DPF domain.

The importance of the multifunctional DPF domain is further demonstrated by the mislocalization of Eps15R when this domain is deleted (figure 3*g*). In contrast to the presence of GFP-Eps15R in both the nucleus (figure 1*d*) and punctate cytoplasmic foci (figure 1*f*), the latter aspect of localization is not seen upon expression of the GFP-Eps15R-*Δ*DPF construct; instead there is reticulated expression throughout the cytoplasm, while the nuclear localization is retained (figure 3*g*). As the appendages of the endocytic adaptor α-adaptin are known to interact with DPF domains of endocytic proteins such as Eps15, epsin and AP180 [24], the loss of this region of Eps15R may inhibit its endocytic compartmentalization.

In contrast to its ability to activate BMP-responsive target genes, the DPF domain antagonized the TGF-β pathway, preventing induction of the dorsal mesodermal markers *chordin*, *goosecoid* and *Frzb* in response to Smad2 (figure 3*h*). This suggests that Eps15R modulates the two major branches of the TGF-β superfamily, enhancing Smad1 signalling while antagonizing the Smad2 pathway.

### 3.4. Eps15R localizes to distinct cellular compartments when complexed with different Smads

To ask whether Smad1 interacts with Eps15R in a specific sub-cellular compartment, we performed bimolecular fluorescence complementation (BiFC), a technique that allows one to observe protein associations in living cells [26,27]. In this approach, one protein is tagged with the N terminal half of the YFP variant Venus and the other with the C terminal half: if the two halves are brought together by interaction between Smad1 and Eps15R, they form a functional fluorophore.

**Figure 4.**
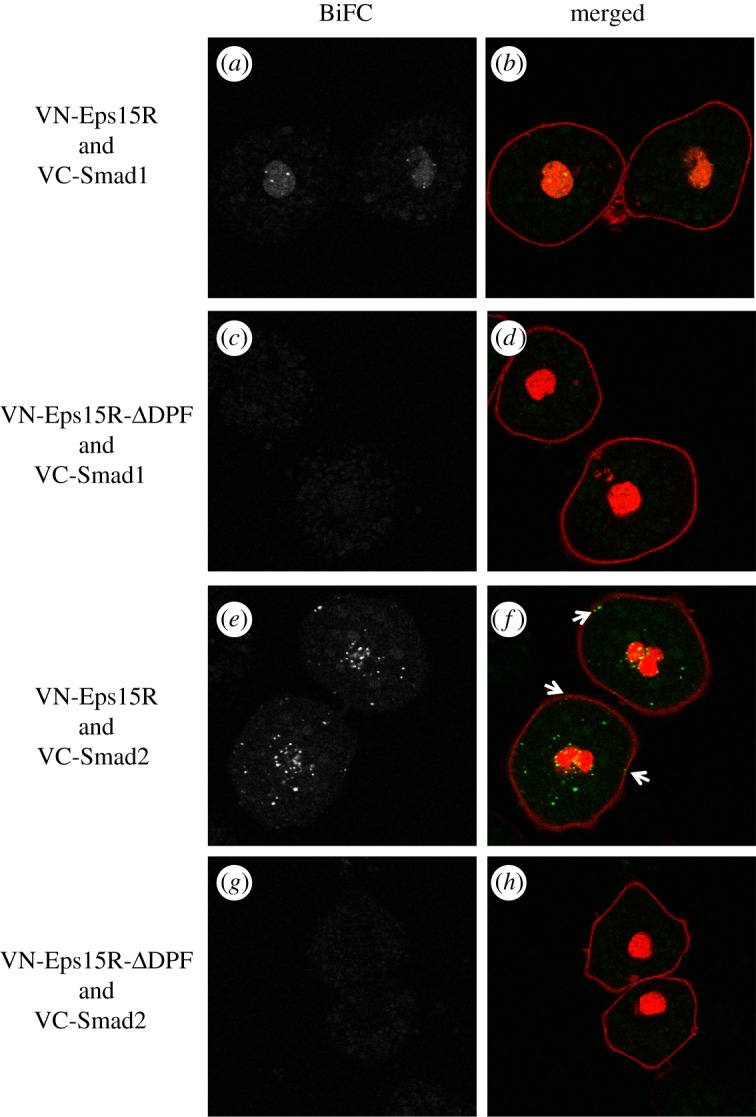
Differential compartmentalization of Eps15R/Smad complexes. Live imaging of Eps15R/Smad complexes monitored by bimolecular fluorescence complementation (BiFC). (*a,c,e,g*) Greyscale images of BiFC fluorescence. (*b,d,f,h*) Merged images of BiFC fluorescence in green and CFP-histone H2B (to label nucleus) and CFP-GPI (to label membranes) in red. Total numbers of cells scored for each BiFC complex were: *n* = 78 (Eps15R/Smad1); *n* = 27 (Eps15R-*Δ*DPF /Smad1); *n* = 45 (Eps15R/Smad2); *n* = 24 (Eps15R-*Δ*DPF/Smad2). (*a,b*) Cells injected with VN-Eps15R and VC-Smad1 have nuclear BiFC fluorescence, with enrichment in localized regions. (*c,d*) VN-Eps15R-*Δ*DPF does not interact with VC-Smad1. (*e,f*) Complexes of VN-Eps15R and VC-Smad2 are distributed in punctate dots throughout the cell. Some complexes are closely associated with the membrane (arrows), consistent with the known association of Eps15R with coated pits. (*g,h*) VN-Eps15R-*Δ*DPF does not interact with VC-Smad2.

Nuclear fluorescence was seen in isolated *Xenopus* ectodermal cells after co-injection of VN-Eps15R and VC-Smad1 RNAs (figure 4*a*,*b*). Complexes were present in intense punctate foci, suggesting that the proteins function within a nuclear sub-compartment. This result, in combination with our finding that Eps15R enhances Smad1 signalling and transactivates gene expression, suggests that Eps15R acts as a component of the Smad1 transcriptional complex. No fluorescence was detected outside the nucleus, indicating that Eps15R does not localize with the cytoplasmic fraction of Smad1 and that Smad1 does not associate with Eps15R near the cell membrane. Deletion of the DPF domain prevented the interaction of Eps15R with Smad1 (figure 4*c*,*d*), confirming the importance of this domain in facilitating the interaction and serving as a specificity control for BiFC. As GFP-Eps15R-*Δ*DPF bears the identical R589* mutation yet is still expressed in cells (figure 3*g*), this point mutation does not render the truncated protein unstable.

Our discovery that the Eps15R DPF domain abrogates Smad2 signalling (figure 3*h*), yet mimics Smad1 activity, inspired us to investigate further the relationship between Eps15R and Smad2 using BiFC. Complexes of Eps15R and Smad2 were detected by BiFC (figure 4*e*,*f*); this interaction also requires the DPF domain (figure 4*g*,*h*). Intriguingly, the localization of Eps15R/Smad2 complexes differs from that of Eps15R/Smad1 complexes, being distributed in punctate foci in the cytoplasm, as if in an endocytic vesicle. Additional fluorescent foci were observed adjacent to the cell membrane (figure 4*f*, arrows). These may represent coated pits, because mammalian Eps15R is enriched in this subcellular compartment [28,29]. In some cells, there appears to be a greater prevalence of Eps15R/Smad2 foci in the vicinity of the nucleus (20%; *n* = 9/45) but this is not a consistent observation.

## 4. Conclusions

We show that Eps15R is required for BMP signalling in animal caps, providing the first functional link between this component of the endocytic machinery and the BMP pathway. In drawing this conclusion, we have performed protein–protein interaction studies and loss-of-function and gain-of-function experiments. We note that our experiments have primarily involved *ex vivo* assays, so the importance of Eps15R in modulating BMP signalling during embryogenesis remains to be established, because it can also modulate other embryonic signalling pathways, such as EGF signalling.

The extent to which Eps15R function is conserved in the regulation of BMP and EGF signalling is unknown, but both EGFR and BMPRII can be internalized through clathrin-dependent and clathrin-independent endocytosis and Eps15R has been implicated in both processes in EGFR trafficking [13,30]. Eps15, Eps15R and epsin contain ubiquitin-interacting motifs that bind to ubiquitylated EGFR and are required for clathrin-independent endocytosis [13]. BMPRII can also be ubiquitylated, and Eps15R was identified as a binding partner of BMPRII [30], so it may also be involved in the internalization of this receptor.

Although Eps15R is found in coated pits, the distribution of Eps15R/Smad1 BiFC fluorescence does not resemble any endocytic compartment but is localized to the nucleus. Eps15R may thus have a transcriptional role in BMP signalling, enhancing the weak transcriptional activity of Smad1. The subnuclear distribution of Eps15R/Smad1 complexes is intriguing. Subnuclear structures include PML bodies and transcription factories; it is possible that Eps15R/Smad1 is incorporated in such structures.

The differences in Eps15R distribution, depending upon whether it is associated with Smad1 or Smad2, suggest that the protein has different roles in TGF-β and BMP signalling. The location of the Eps15R/Smad2 complexes is consistent with their incorporation into endocytic vesicles; the family member Eps15 has been detected in early and late endosomes [31]. As the DPF domain of Eps15R attenuates Smad2 signalling, perhaps it represses Smad2 transcriptional complexes, or targets Smad2 for endocytic degradation via the ubiquitin ligase Nedd4L [32].

Smad proteins mediate intracellular antagonism between the activin and BMP pathways in *Xenopus* [33]. One suggested mechanism for such intracellular antagonism is the competition of pathway-specific Smads such as Smad1 and Smad2 for a limited pool of the common Co-Smad, Smad4 [33]. It is possible that endocytic components such as Eps15R may modulate the availability of Smad1 and Smad2 for partnership with Smad4 through differential subcellular targeting via endocytic pathways.

As Eps15R interacts with both BMP and TGF-β receptor Smads (R-Smads), it is likely also to partner with the other R-Smads in these classes: specifically the BMP-Smads, Smad5 and Smad8, and the TGF-β-Smad, Smad3. It will be interesting to determine whether Eps15R can also interact with the more divergent Co-Smad, Smad4, or the inhibitory-Smads, Smad6 and Smad7 [34], and the extent to which the Eps15R/Smad interactions are regulated by Smad phosphorylation status. Smad4 enters into heteromeric signalling complexes with activated R-Smads [35,36], and Eps15R may be associated with these heteromeric complexes; alternatively it may interact with R-Smads in a Smad4-independent manner.

Our work identifies interactions between Eps15R and Smads 1 and 2, and emphasizes the utility of BiFC for investigating Smad signalling, allowing live visualization of spatially distinct Smad compartments that cannot be distinguished with cross-reacting antibodies. It will be interesting to discover whether other DPF domain-containing proteins bind Smads, as this could allow selective trafficking of these important signal transducers by numerous endocytic proteins containing DPF or structurally related NPF domains.

## 5. Methods

### 5.1. Cloning of *Eps15R* genes and construction of expression plasmids

A partial cDNA isolated from a yeast two-hybrid screen using Smad1 as bait [37] was used to screen a *Xenopus* oocyte cDNA library to obtain a full-length *Xenopus* Eps15R cDNA clone (pBSK-Eps15R; Genbank accession no. AY254055). Expression constructs were cloned in the CS2+ vector or its derivatives (see text).

### 5.2. Embryo manipulation and embryonic assays

Embryo culture, isolation and staging were performed under a Home Office licence, as described [21]. Total RNA was prepared from animal caps and analysed by RT-PCR either by Lightcycler (Roche) or by conventional radioactive methods. The Eps15R MO sequence was: 5′-TGAGAGGGATGAGCGCCGCCATCTT-3′; the control was the standard MO from Gene Tools. RT-PCR and confocal results were replicated in independent biological experiments using *n* = 10 animal caps for each sample. *n* > 12 embryos were scored in the morphological assays.

### 5.3. Immunoprecipitation

Embryos were lysed in Oocyte Lysis Buffer (250 mM sucrose, 100 mM NaCl, 2.5 mM MgCl_2_, 10 mM NaF, 10 mM EGTA, 1 mM Na_3_VO_4_, 20 mM HEPES (pH 7.2) and 1% Triton X-100) containing protease inhibitors (10 μg ml^–1^ leupeptin, 1 μg ml^–1^ aprotinin, 1 μg ml^–1^ pepstatin A), cleared by centrifugation and immunoprecipitated with an anti-Flag monoclonal antibody (Sigma) in combination with protein G-Sepharose (Pharmacia) prior to immunoblotting.

### 5.4. Yeast assays

Amino acids 1–253 of *Xenopus* Smad1 were fused to the GAL4-DNA binding domain in plasmid pGBT9. *Xenopus* Eps15R constructs were linked to the yeast GAL4 activation domain in pGAD10. These included a full-length fusion protein (pGAD/full-length), the N-terminal amino acids 1–597 (pGAD/597) and a C-terminal DPF segment encoding amino acids 590–897 (pGAD/DPF). Recombinant plasmids were co-transformed into the yeast reporter strain Y153, subjected to HIS3^−^ selection and scored for β-galactosidase activity to confirm protein–protein interactions. To test for Eps15R intrinsic transcriptional activity, amino acids 1–597, 1–365 and 590–897, corresponding to the Eps15R 597, EH and DPF segments respectively, were fused to the GAL4 DNA-binding domain and cloned in pGBT9.

### 5.5. Bimolecular fluorescence complementation and confocal microscopy

BiFC constructs were tagged N-terminally with optimized versions of the N-terminal or C-terminal fragments of Venus [38]. ORFs were fused in frame to the BiFC fragments via a 6-Arg linker to generate VN-Eps15R and VC-Smad1; VC-Smad2 has been described [38]. Site-directed mutagenesis of Eps15R (R589*) was used to make VN-Eps15R-*Δ*DPF as well as the GFP-Eps15R-*Δ*DPF used in localization experiments. Animal caps from embryos co-injected with 100 pg each of VN-Eps15R RNA and either VC-Smad1 or VC-Smad2 were dissociated into single cells by cutting and culturing caps from NF8 embryos in 1 × CMFM solution (88 mM NaCl, 1 mM KCl, 2.4 mM NaHCO_3_, 7.5 mM Tris, pH 7.6) for 30 min. The unpigmented inner epithelial cells were separated from the outer pigmented cells as the latter are refractory to dissociation. The inner epithelial cells were transferred onto glass-bottomed dishes (MatTek Corporation), pre-coated with E-cadherin, and containing ¾ NAM and 0.2 per cent BSA. The dishes were subjected to minimal movement for 30 min to allow the cells to adhere before they were imaged live by confocal microscopy [39].

## Supplementary Material

Supplementary Figure 1

## Supplementary Material

Supplementary Figure 2
