# Supplementary material for: Eps15R is required for bone morphogenetic protein signalling and differentially compartmentalizes with Smad proteins
Source: Open Biol. 2012 Apr;2(4):120060. doi: 10.1098/rsob.120060 (PMC3376731; doi:10.1098/rsob.120060)
Supplement: Supplementary Figure 1 [file rsob120060-s1.pdf]

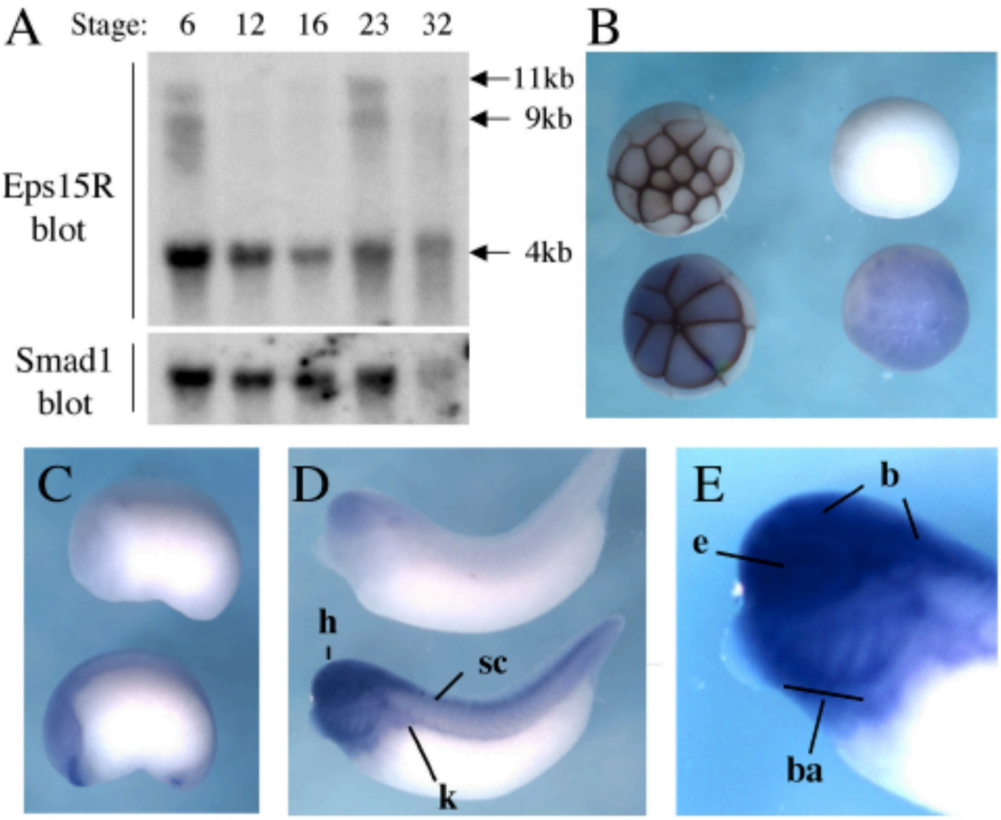

Supplementary Figure 1: Expression of *Xenopus* Eps15R during embryonic development. (A) A Northern blot analysis shows that Eps15R is expressed at all stages of early development. Embryonic stages correspond to blastula (NF6), late gastrula (NF12), early neurula (NF16), early tadpole (NF23) and tailbud tadpole (NF32). Smad1 expression is unchanged over early embryogenesis and serves as a loading control. (B, C and D) Whole-mount in situ hybridization of Eps15R in developing embryos. (B) At blastula stages Eps15R transcripts are present in the animal pole. The top right embryo was stained with sense probe for background staining. (C) In the neurula, Eps15R expression is enriched in the neural folds, cement gland and proctodeum. (D) In the early swimming tadpole (NF36) Eps15R is expressed in head (h), spinal cord (sc) and kidney (k). The upper embryo in panels C-D is a negative control (sense probe) for background staining. (E) Close up of the head of the lower tadpole in panel D showing expression in the eye (e) brain (b) and branchial arches (ba).
