# Supplementary material for: Eps15R is required for bone morphogenetic protein signalling and differentially compartmentalizes with Smad proteins
Source: Open Biol. 2012 Apr;2(4):120060. doi: 10.1098/rsob.120060 (PMC3376731; doi:10.1098/rsob.120060)
Supplement: Supplementary Figure 2 [file rsob120060-s2.pdf]

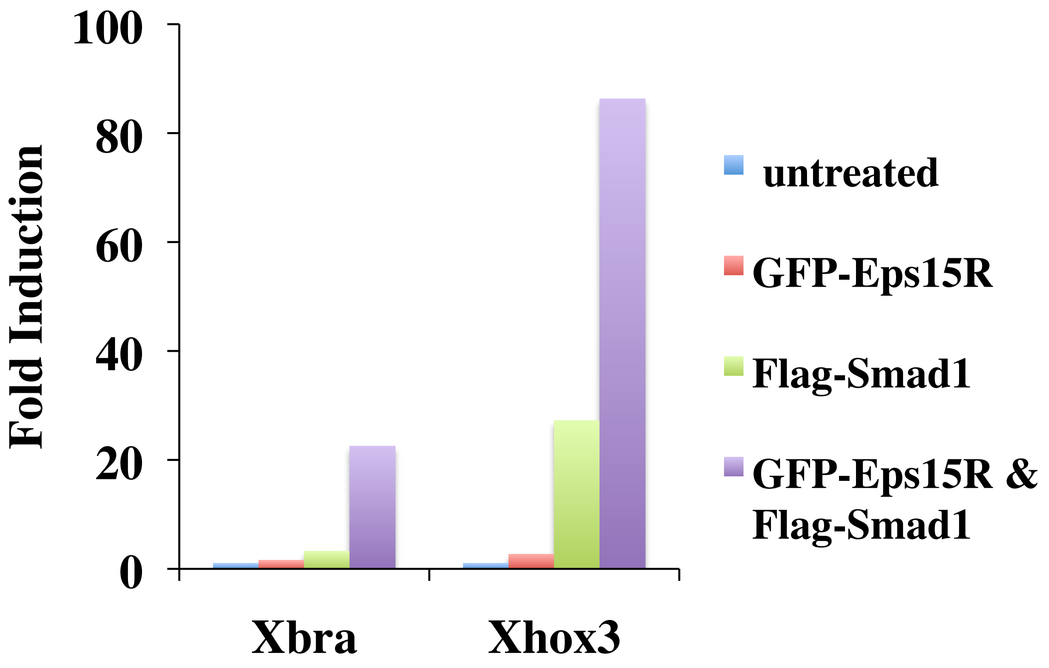

Supplementary Figure 2: Synergism of GFP-Eps15R with Smad1

GFP-Eps15R mRNA replicates the activity of untagged Eps15R mRNA shown in Fig. 2(h), synergising with Flag-Smad1 to induce the BMP targets Xbra and Xhox3 in animal caps. 2ng of each RNA were injected and the caps were harvested at NF11.5.
